# Proteomimetics of Natural Regulators of JAK–STAT Pathway: Novel Therapeutic Perspectives

**DOI:** 10.3389/fmolb.2021.792546

**Published:** 2022-01-03

**Authors:** Sara La Manna, Ilaria De Benedictis, Daniela Marasco

**Affiliations:** Department of Pharmacy, University of Naples “Federico II”, Naples, Italy

**Keywords:** JAK–STAT pathway, SOCS proteins, inflammation, proteomimetics, feedback regulators

## Abstract

The JAK-STAT pathway is a crucial cellular signaling cascade, including an intricate network of Protein–protein interactions (PPIs) responsible for its regulation. It mediates the activities of several cytokines, interferons, and growth factors and transduces extracellular signals into transcriptional programs to regulate cell growth and differentiation. It is essential for the development and function of both innate and adaptive immunities, and its aberrant deregulation was highlighted in neuroinflammatory diseases and in crucial mechanisms for tumor cell recognition and tumor-induced immune escape. For its involvement in a multitude of biological processes, it can be considered a valuable target for the development of drugs even if a specific focus on possible side effects associated with its inhibition is required. Herein, we review the possibilities to target JAK–STAT by focusing on its natural inhibitors as the suppressor of cytokine signaling (SOCS) proteins. This protein family is a crucial checkpoint inhibitor in immune homeostasis and a valuable target in immunotherapeutic approaches to cancer and immune deficiency disorders.

## Introduction

Janus tyrosine kinases (JAKs) play crucial roles in the transduction of signals triggered by membrane receptors of interleukins during inflammatory response (Groner and von Manstein, 2017; Bousoik and Montazeri Aliabadi, 2018). They are associated with important downstream proteins including signal transducers and activators of transcription (STATs), which in turn regulate the expression of a variety of proteins involved in the induction/prevention of apoptosis (Loh et al., 2019). STATs are able to induce transcription and epithelial/mesenchymal transition (EMT), affect gene expression, generate a pro-tumorigenic microenvironment, and promote cellular self-renewal and differentiation and are involved in the formation of pre-metastatic niches (Groner and von Manstein, 2017; Piranlioglu et al., 2019). Through the JAK-STAT pathway, cytokines, interferons (IFNs), and growth factors are regulated, and extracellular signals are converted into programs of cell growth and differentiation (Yan et al., 2018). It is essential in innate and adaptive immune responses, and its aberrant activation was highlighted in neuroinflammatory diseases, tumor cell recognition, and tumor-driven immune escape (Owen et al., 2019). In addition, the involvement in proliferation and survival provides important roles in resistance to targeted drug treatments (Nascimento et al., 2017).

Aberrant activation of JAK-STAT is encountered in many immune-mediated diseases (Jamilloux et al., 2019), including rheumatoid arthritis (RA) (Malemud, 2018), systemic lupus erythematosus (SLE) (Goropevšek et al., 2017), psoriatic arthritis (PsA) (Fiocco et al., 2014), psoriasis (Kwatra et al., 2012), inflammatory bowel disease (IBD) (Salas et al., 2020), Crohn’s disease, ulcerative colitis (Rogler, 2020), discoid lupus erythematosus (DLE), and dermatomyositis (DM) (Kahn et al., 2018). The hyperactivation of JAK-STAT has been highlighted in the aforementioned diseases, but more recently, it has also been reported in patients with pulmonary hypertension (PH): in their isolated pulmonary arteries, JAK2 and STAT3 mRNA transcript levels and protein expressions were higher than those in health subjects (Roger et al., 2021). In cancer, the hyperactivation of this pathway can be either due to a JAK mutation, as mostly observed in some hematological malignancies (Vainchenker and Constantinescu, 2013), or due to the intrinsic nature of cancer, as in some solid tumors (Qureshy et al., 2020). The activation of the JAK-STAT pathway is strictly correlated to the expression of pro-inflammatory genes, and it is linked to the formation of atheromatous plaques. During early stages of atherogenesis, in vascular smooth muscle cells (VSMCs) and mice models of atherosclerosis, the leukocyte recruitment, the migration and proliferation of VSMCs, and the formation of lipid-laden macrophages occur combined with the activation of the pathway (Gharavi et al., 2007; Boengler et al., 2008; Lim et al., 2008; Ortiz-Muñoz et al., 2009; Taleb et al., 2009; Qin et al., 2014), which, in turn, attenuates nicotinamide adenine dinucleotide phosphate (NADPH) oxidase (NOX) expression and activity (Manea S. A et al., 2010; Manea A et al., 2010; Fenyo et al., 2011).

The plethora of involved diseases suggests that the employment of drugs that are able to downregulate JAK-STAT could have beneficial effects (Favoino et al., 2021). The JAK family is made up of four kinases: JAK1-3 and TYK2. While JAK1, 2, and TYK2 are ubiquitously expressed in mammals, JAK3 is expressed mainly in hematopoietic and lymphoid tissues (Ghoreschi et al., 2009). JAKs share a common modular structure with seven JAK homology (JH) regions including the catalytically active kinase domain (JH1) and pseudokinase domain (JH2) without catalytic activity. JAK mechanisms are tuned by their phosphorylation states through transphosphorylation events. JAKs, in turn, phosphorylate tyrosines of type I/II cytokine receptors and STATs, inducing their dimerization and translocation into the nucleus for transcription of pro-inflammatory genes (Rawlings et al., 2004). STATs consist of seven DNA-binding proteins: STAT1-6 (5A, 5B variants) regulate the effector responses, STAT1, 4 are responsible for the antiviral type 1 (Th1) response, STAT6 is responsible for the anti-helminth type 2 (Th2), and STAT3 is responsible for the antibacterial/fungal type 3 (Th17) (O’Shea et al., 2015). Conversely, STAT5 induces the expression of the T-regulatory cell (Treg) transcription factor, Foxp3, which is implicated in their differentiation (Burchill et al., 2007). In the present review, different from many others concerning the inhibition of the JAK-STAT pathway with small-molecule ATP-competitive inhibitors, we focused on the structural approach to design modulators of the entire pathway as mimetics of natural inhibitors as SOCS proteins are natural inhibitors of the kinase activity of JAKs.

## Molecular Basis of JAK-STAT Regulation: Natural Inhibition

**FIGURE 1:**
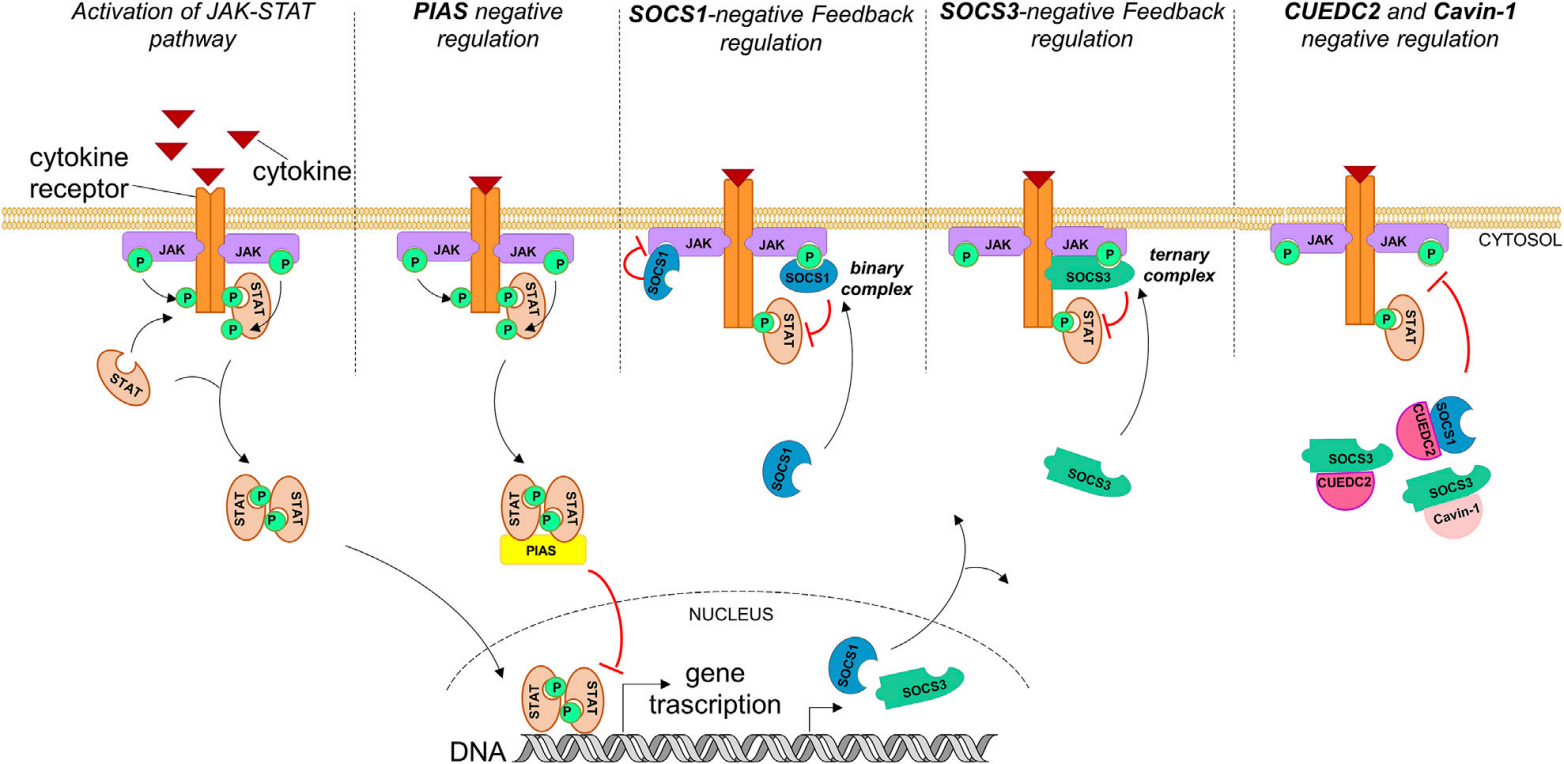
Endogenous activation of the JAK-STAT pathway and its regulation through natural inhibitors. PIASs act mainly by suppressing STAT-regulated gene expression and inhibiting STAT binding to DNA. Hyperactivation of the JAK-STAT pathway also activates its own negative feedback regulators: SOCS proteins. SOCS1 and SOCS3 inhibit signaling by different mechanisms: SOCS1 forms a binary complex with JAKs (phosphorylated or unphosphorylated) and inhibits their catalytic activity; SOCS3 inhibits JAK activity through the formation of a ternary complex, binding simultaneously to the cytokine receptor and JAKs. Cavin-1 and CUEDC2 inhibit the JAK-STAT pathway by interacting with SOCS1,3 via an SH2 domain PEST sequence.

Suppressors of cytokine signaling (SOCS) proteins negatively regulate JAK-STAT and consist of eight members: SOCS1-7 and cytokine-inducible SH2-containing protein (CIS) (Alexander and Hilton, 2004; Croker et al., 2008; Yoshimura et al., 2007). While CIS and SOCS1,3 are well characterized as negative feedback regulators of this pathway, the biological functions of other members are still under investigation (Linossi et al., 2013). SOCS proteins have a modular organization of regions: 1) an N-terminal region of variable length, 2) a central Src homology 2 (SH2) domain, and 3) a well-conserved C-terminal domain named SOCS box (Yoshimura et al., 1995). Only SOCS1 and 3 bear a kinase inhibitory region (KIR) in the N-terminal part, even with different mechanisms of action (MOAs). Indeed, while SOCS3 binds simultaneously to JAK and the cytokine receptor proteins (Babon et al., 2014), SOCS1 binds to JAK even in its unphosphorylated form (Liau et al., 2018) (as schematically described in Figure 1) and demonstrates the most potent inhibitor independently from the presence of receptors (Nicholson et al., 1999).

SOCS proteins are induced individually by cytokines/growth factors, generating a negative feedback loop with a strictly regulatory function in cellular development and homeostasis, and often demonstrate tumor suppressor and anti-inflammatory effects (Trengove and Ward, 2013) since the repression of SOCS1, 3 leads to the progression of inflammatory diseases (Xin et al., 2020). Hyperglycemia contributes to renal damage through hyperactivation of JAK-STAT in diabetic nephropathy; it has been demonstrated that the overexpression of SOCS proteins reverts the glucose-induced activation. The intrarenal delivery of adenovirus expressing both SOCS1 and SOCS3 in diabetic rats ameliorated renal function and reduced lesions associated with diabetes, as well as the activation of STAT1 and STAT3 and the expression of pro-inflammatory and profibrotic proteins in the diabetic kidney (Ortiz-Muñoz et al., 2010).

In infections caused by herpes simplex virus, cytomegalovirus (Alston and Dix, 2019), chronic hepatitis B virus (HBV), and hepatitis C virus (HCV) (Xie et al., 2021), overexpression of SOCS1 and 3 was observed: this behavior is likely due to an escape mechanism to SOCS-dependent immune regulation. In this context, the employment of inhibitors of SOCSs could provide therapeutic effects (Akhtar and Benveniste, 2011).

SOCS1 strictly regulates IFN signaling (Alexander et al., 1999; Fenner et al., 2006), and interleukins (IL)-2 (Davey et al., 2005), -12, -23 (Eyles et al., 2002), -6 (Tamiya et al., 2011) and their overexpression repress pro-inflammatory genes such as intercellular cell adhesion molecule-1 (*ICAM-1*), C-X-C motif chemokine ligands (*CXCL*) 9,10, and C-C motif chemokine ligands (*CCL*) 2, 5 (Madonna et al., 2008). Its deficiency leads to inflammation (Ihle et al., 1995; Ilangumaran et al., 2004), whereas its haploinsufficiency induces autoimmune diseases (Hadjadj et al., 2020). The ROS-dependent apoptosis of immune cells induces SOCS1 expression, and conversely, SOCS1 overexpression represses apoptosis caused by oxidants (Jung et al., 2016; Schuett et al., 2019). In atherosclerotic models, the inhibition of JAK2, STAT1, and STAT3 prevented lesion formation (Torella et al., 2007; Miklossy et al., 2013), and many efforts based on the therapeutic role of SOCS1,3 effectively deregulate pathological JAK-STAT hyperactivation in cardiovascular disease studies (Recio et al., 2015; Lopez-Sanz et al., 2018).

There is a direct interaction between p53 and SOCS1 that involves the SH2 domain of SOCS1 and the p53 N-terminal transactivation domain 2 (TAD2). Recent investigations outlined that SOCS1 can be phosphorylated on Tyr^80^ in its extended SH2 domain (ESS) by members of the SRC family of non-receptor tyrosine kinases (SFKs) (Saint-Germain et al., 2019). The phosphorylation modification makes SOCS1 able to dimerize. Both phosphorylation and dimerization regulate the p53-SOCS1 tumor suppressor axis: SOCS1 bridges p53 and ATM at DNA damage foci, leading to p53 phosphorylation and a subsequent increase in its transcriptional activity (Calabrese et al., 2009).

During early phases of atherosclerosis, STAT3 activation represses SOCS3 levels that, in turn, increases resistin, cytokine, and reactive oxygen species (ROS) production depending on NADPH oxidase (by increased NOX1 expression) (Gan et al., 2013; Barrett et al., 2019), while the overexpression of SOCS1 represses ROS generation by inhibiting NOX expression caused by the upstream inactivation of JAK2, STAT1, and phosphoinositide 3-kinase (PI3K) (Hernández-Vargas et al., 2005; Ortiz-Muñoz et al., 2009; Recio et al., 2015). Physiological levels of SOCS3 are unable to limit acute inflammation (Orr et al., 2007), but the presence of its recombinant form suppresses it (Jo et al., 2005). Indeed, in malignant fibrous histiocytoma (Shouda et al., 2006), colorectal (Chu et al., 2017), ovarian cancer cell lines (Shang et al., 2017), and solid tumors based on the IL-6/STAT3 axis, SOCS3 overexpression demonstrated beneficial effects: in T47D and in MCF7 BC (breast cancer) cells, it represses cell proliferation through reduction in STAT3 expression and STAT5 phosphorylation (Barclay et al., 2009), and in mouse xenograft models, it represses tumor growth and metastasis formation (Hill et al., 2010), while downregulated SOCS3 expression provides enhanced risks of recurrent disease in BC patients (Ying et al., 2010). On the other hand, STAT3 hyperactivation [as in cholangiocarcinoma (CCA)] determines an epigenetic silencing of the SOCS3 gene and cellular resistance to apoptosis and induction of EMT (Isomoto, 2009). In this context, a SOCS3 mimetic could have great therapeutic importance since, in addition to being a potential anti–IL-6R compound, it could revert harmful effects of STAT3 activation for the progression of carcinoma (Zhou et al., 2015).

Recently, several pieces of evidence emerged of additional SOCS-interacting proteins required for optimal inhibition of cytokine signaling: cavin-1, an essential component of caveolae (Cheng and Nichols, 2016), and CUEDC2 [coupling of ubiquitin to endoplasmic reticulum degradation (CUE) domain-containing protein 2], which is important for cell cycle control (Gao et al., 2011; Zhang et al., 2013). Their MOAs are schematically reported in Figure 1. In detail, the interaction of SOCS3 with cavin-1 requires its SH2 domain and is independent of its ability to bind Tyr-phosphorylated targets. The structural required element for the interaction is a PEST sequence present in almost all SH2 domains (Wang et al., 2001); the SOCS3 PEST sequence revealed that it is able to control its turnover without affecting the inhibition of JAK-STAT (Babon et al., 2006). Conversely, CUEDC2 can suppress the phosphorylation and activation of the JAK1-STAT3 axis but requires the presence of SOCS3 for a similar direct interaction assessed *in vitro* (Zhang et al., 2012). However, at present, no structures of SOCS3 or its SH2 domain with either full length cavin-1 (Williams et al., 2018) or CUEDC2 are available (Zhang et al., 2012). The lack of structural information prompted several authors to employ an overlapping peptide array approach with the aim to identify and optimize high-affinity SOCS-derived sequences as antagonists of the interaction (Brown et al., 2013). The identification of CUEDC2 and cavin-1 as regulators of SOCS function and stability could represent another potent approach for the modulation of the JAK-STAT pathway, since both cavin-1 and CUEDC2 enhance SOCS3 function (and SOCS1 in the case of CUEDC2).

An important class of protein regulators of STAT proteins that function often in conjunction with SOCS is represented by the protein inhibitor of activated STAT (PIAS) (Niu et al., 2018). In RA pathology, SOCSs and PIASs are dysfunctional (Malemud, 2016), and experimental overexpression of SOCS3 in RA synovial tissue represses the inflammation (Shouda et al., 2001; Mahony et al., 2016; Silvagni et al., 2021). Different from SOCSs, they are expressed constitutively and are involved in apoptosis during normal homeostasis, as well as cell survival and tissue renewal (Mohan et al., 2020; Xin et al., 2020). They belong to a protein family of five components: PIAS1, PIASxα, PIASxβ, PIAS3, and PIAS4 (PIASγ) (Heppler and Frank, 2017), with a modular structure having a serine/threonine-rich domain located at the C terminus, a Zn-binding RING finger–like domain at the central portion, and a conserved SAP domain (scaffold attachment factor A/B, Acinus, PIAS) close to the N terminus (Kipp et al., 2000). PIAS proteins inhibit STAT transcriptional activity through three major mechanisms: 1) the direct interaction with STAT and blocking of STAT–DNA interactions (Prigge and Schmidt, 2006), 2) the recruitment of transcriptional cofactors to STAT target genes (Heppler and Frank, 2017), and 3) the induction of SUMOylation of the protein (Ungureanu et al., 2003; Yuan et al., 2015) (Figure 1).

## Inhibition of JAK-STAT Signaling: Small Molecule Approach

Currently, the employment of inhibitors of JAK proteins as small molecules is clinically accepted in proliferative neoplasia and in autoimmune diseases, where they modulate immune responses, T-cell differentiation and B-cell activation (Villarino et al., 2015). Conversely, they are under clinical investigation in chronic inflammatory skin diseases where they can be also be applied topically with innovative formulation systems (Welsch et al., 2017). Usually, JAK inhibitors (JAKis) (Table 1) target the ATP-binding site of the kinase domain but simultaneously block different pathways: these features confer to these agents a “broad-spectrum” MOA (Qureshy et al., 2020) as well as immunosuppressive drugs (Flanagan et al., 2010). Preclinical studies in solid tumor cell lines show that JAK inhibitors decrease STAT activation, cell proliferation and survival, and tumor growth in *in vivo* models (Schwartz et al., 2017). The first U.S. Food and Drug Administration (FDA)–approved JAKi, ruxolitinib, is a JAK1,2 inhibitor that was registered for myeloproliferative disorders (Verstovsek et al., 2010); during preclinical studies, it demonstrated the ability to sensitize cell lines and murine models to chemotherapy, immunotherapy, and oncolytic viral therapy and provides good results in combination with other drugs as capecitabine for advanced/metastatic pancreatic cancer (Hurwitz et al., 2018) and BC (O'Shaughnessy et al., 2018) or afatinib in non–small-cell lung carcinoma (NSCLC) (Park et al., 2019). In multiple myeloma (MM) patients’ cells and in RPMI-8226 and U266 MM cell lines, JAK1 and JAK2 resulted in overexpression, and upon treatment with ruxolitinib and bortezomib, these cells presented 50% of cells in late apoptosis, a reduction in antiapoptotic gene expression, and a higher number of cells in the SubG0 phase. In addition, the combination of ruxolitinib, bortezomib, and lenalidomide provided 72% of cell death similar to the results obtained upon the treatment of a combination of bortezomib, lenalidomide, and dexamethasone that is actually employed in clinical practice (de Oliveira et al., 2017). Tofacitinib, selective for JAK2, 3, was approved by the FDA for the therapy of RA and ulcerative colitis (Dhillon, 2017), while in IBD, it demonstrated efficacy in early phase trials (Harris and Cummings, 2021).

**TABLE 1:** Most common JAKis: their preferential targets and diseases where they are on market/in clinical trials.

Name	Selectivity	Diseases
Ruxolitinib	JAK1, 2	Myelofibrosis; polycythemia vera; essential thrombocythemia; myelodysplastic syndrome; acute and chronic leukemias; MM; B-cell and Hodgkin’s lymphoma; prostate, pancreatic, and BC; psoriasis; RA
Tofacitinib	PAN JAK	Rheumatoid psoriatic and juvenile idiopathic arthritis, ankylosing spondylitis, dry eye, renal transplant, ulcerative colitis, psoriasis, dermatitis
Baricitinib	JAK1,2	RA, diabetic kidney disease, autoinflammatory syndromes
Lestaurtinib	AK1/JAK2,3, FLT3, TrkA/B/C	Myelofibrosis, neuroblastoma, psoriasis, MM
Pacritinib	JAK1, FLT3	Myelofibrosis, myelodysplastic syndrome, leukemia, lymphoma
Momelotinib	JAK1,2, CDK2	Myelofibrosis, pancreatic cancer
Fedratinib	JAK2	Myelofibrosis, solid tumors, renal impairment, hepatic impairment
Filgotinib	JAK1	RA, Crohn’s disease, urinary incontinence
Decernotinib	JAK1,2	RA
Gandotinib	JAK2	Myelofibrosis
AZD1480	JAK2	Myelofibrosis, solid tumors

One of the most important issues in BC is the onset of chemoresistance. A recent study reported the effects of several JAKis on the kinase activity of chemotherapy-resistant MCF7 (MCF7Res) and chemotherapy-sensitive MCF7 cells. In detail, the treatment with tofacitinib in combination with doxorubicin induced a conversion from chemoresistant to chemosensitive cells, leading to apoptosis (Nascimento et al., 2017). In Table 1, most common JAKis and their preferential targets and diseases for which they are on the market/in clinical trials are reported.

While JAKis are in advanced stages of clinical development, inhibitors of STATs are in early development since these proteins are less easily druggable with respect to enzymes such as JAKs. STAT3 and STAT5 demonstrated to be involved in many cellular processes including the regulation of growth and survival (de Araujo et al., 2020); consequently, they also exert a primary role in oncological processes. Efforts in the inhibition of STATs include small molecules, antisense and decoy oligonucleotides, and oligopeptides (Gadina et al., 2018; Yang et al., 2019).

Phosphorylated STAT3 (pSTAT3) is strictly involved in cellular plasticity processes that imply the coexistence of cancer stem cells (CSCs) and the EMT. The administration of a STAT3 inhibitor, napabucasin, revealed its ability to improve chemosensitivity of resistant cells acting at the level of interconversion from differentiated to stem-like states (Shih and Mei, 2021).

A series of non-competitive inhibitors of STAT3 were identified by structure-based high-throughput virtual screening; it includes STA-21, Stattic (Stat three-inhibitory compound), S3I-201/NSC74859, BP-1–102, OPB-31121, TTI-101 (C188-9), and their analogs. Most of them demonstrated the ability to inhibit cell growth of several cancer lines for the suppression of the binding of STAT3 to its pY-peptide ligands and thus blocking IL-6–STAT3 activation, nuclear translocation, and transcriptional gene activation. Preclinical studies of these small STAT3 inhibitors showed promising results (Bharadwaj et al., 2020): when tested in several mouse models of fibrosis, including bleomycin-induced lung and skin fibrosis (scleroderma) and in combination with cucurbitacin-B, some of them revealed their ability to reduce carbon tetrachloride–induced liver fibrosis that is accompanied by reduced levels of pSTAT3 (Sallam et al., 2018).

One controversial issue concerns the selectivity of JAKis: first-generation inhibitors target different JAKs, but their efficacy is often associated with adverse events. Indeed, herpes zoster infections were more frequent in patients treated with tofacitinib than in those receiving biological therapeutics such as antibodies siltuximab (anti–IL-6), tocilizumab (anti–IL-6R), secukinumab (anti–IL-17), and mepolizumab (anti–IL-5) (Dymock et al., 2014). In addition, in several cases, thrombocytopenia and neutropenia were encountered (Lee et al., 2014). Nevertheless, similar studies in RA patients did not point out increased risk of hematological cancers or solid tumors after long-term treatment with ruxolitinib (Durham et al., 2019). Actually, many pharmaceutical efforts toward selective JAKis are underway as filgotinib is selective for JAK1 (Mease et al., 2018). However, the exclusive search for selectivity could lead to a loss of inhibitory effects and points out the necessity of novel therapeutic strategies including alternative ways targeting JAK-STAT with minimum adverse reactions (Ou et al., 2021).

## Proteomimetics of Natural Inhibitors of JAK-STAT

Protein–protein interactions (PPIs) tune biological processes and are deeply involved in the development and progression of diseases (Milroy et al., 2014). Proteomic investigations pointed out that hundreds of thousands of PPIs occur (Stumpf et al., 2008); hence, considerable pharmaceutical efforts are focused on their modulation for therapeutic intervention (Barnard et al., 2015; Scott et al., 2016; Feng et al., 2017; Stefan et al., 2018; Hymel et al., 2021). In this context, small-molecule approaches often fail mainly because such a compound for its size cannot establish a suitable number of favorable interactions, thus hampering this traditional pharmaceutical approach. Conversely, proteomimetics represent more efficient and promising agents toward PPIs. In them, a scaffold reproducing the spatial and angular projections of “hot spot” side chains at the protein interfaces (Gigante et al., 2020) mimicking secondary (e.g., α-helix and β-strand) and tertiary (e.g., coiled-coil) structure (de Araujo et al., 2014; Lingard et al., 2014; Di Natale et al., 2020a) is developed. In this context, peptides and peptidomimetics are elective compounds (Scognamiglio et al., 2013; Carotenuto et al., 2014; La Manna et al., 2018a): they act as proteomimetics able to selectively recognize their target protein in biophysical assays and to mimic, totally or partially, the cellular function of native proteins (Lonardo et al., 2010; Causa et al., 2013; Sawyer et al., 2017). A prominent strategy, mostly lying on the knowledge of the structure of protein complex, consists in the identification of isolated protein fragments mainly responsible for the formation of the protein complex and successively on their conformational stabilization through covalent restraints to improve the affinity and stabilities of unstructured peptides for target proteins (Russo et al., 2015; Soler et al., 2017; Adedeji Olulana et al., 2021; La Manna et al., 2021). In this scenario, several structure-based inhibitors demonstrated discrete success. Annexin A1 (AnxA1) protein has a potent anti-inflammatory effect, and its mimetic peptide, Ac2-26, was able to ameliorate inflammation and wrinkle formation in TNF-α/IFN-γ-stimulated human keratinocytes (HaCaT) and fibroblasts (Detroit 551) and decrease the expression of pro-inflammatory chemokines through the inhibition of several cellular pathways including JAK-STAT. It also induced the synthesis of collagen by generating pro-collagen and the suppression of collagen degradation by inhibiting the expression of collagenases matrix metalloproteinase (MMP)-1 and MMP-8 (Kim et al., 2020). Similarly, the N-terminal domain (NTD) of STAT3 is considered to be mainly involved in STAT3-interactomes, and structural data available allowed for the rational design of analogs of the helix 2 of STAT3 able to directly and selectively bind STAT3 but not STAT1 and to induce apoptosis in BC but not in normal breast cells or STAT3-deficient fibroblasts (Timofeeva et al., 2007). By applying the same approach to STAT1, its NTD was employed to design inhibitors of STAT1 transactivation able to block cytokine-induced STAT1-dependent proliferation in metanephric progenitors, tubulogenesis, and several antiapoptotic factors such as myeloid cell leukemia-(Mcl)-1 and heat shock protein (Hsp)-27 in renal tumor cells with constitutively active STAT1 (Wang et al., 2010).

### Mimetics of SOCS Proteins

SOCS proteins have crucial regulatory functions in immune homeostasis, and the reproduction of their biological roles is conceived as a valuable strategy in immunotherapeutic approaches in cancer and immune deficiency disorders (Johnson et al., 2020). In this scenario, new SOCS1, 3 proteomimetics endowed with anti-inflammatory/antioxidant properties have been designed and tested by us and other groups, paving the way to the employment of peptide-based compounds as novel therapeutics that, recently, are under growing magnifying light (Viparelli et al., 2008; Russo et al., 2016; La Manna et al., 2017a; Madonna et al., 2017; La Manna et al., 2018a; La Manna et al., 2018b; La Manna et al., 2020a).

#### SOCS1

SOCS1 is a direct inhibitor of JAKs; from the crystal structure of the complex SOCS1-JAK1, SOCS1 employs both SH2 and KIR domains for the protein interaction, exhibiting poor structural variations once bound to the kinase. Acting as a pseudosubstrate of kinase (Giordanetto and Kroemer, 2003), only KIR appears more ordered with respect to the unbound state, even if it does not assume any canonical secondary structures (Liau et al., 2018). Within it, the fragment His^54^-Arg^59^ forms polar and hydrophobic bonds with residues located in JAK1 catalytic domain. Between the side chains of His^885^ and Pro^1044^ of JAK1, His^54^ of SOCS1 is inserted, while Phe^55^ acts as the P+1 residue of the pseudosubstrate. These residues, along with Phe^58^, form the greatest number of hydrophobic contacts with the activation loop and the α-G helix of kinase (Liau et al., 2018).

On the basis of previous mutational studies, several research groups carried out numerous studies where they demonstrated that the linear peptide spanning KIR domain (52–67) inhibits/reduces 1) the activation of STAT by cytokines Th1 and 17 in leukocytes; 2) the activation and migration of vascular cells and macrophages *in vitro* (Ahmed et al., 2015); 3) the expression of cytokines with pro-inflammatory properties in atherosclerotic plaques (Recio et al., 2014); 4) the renal inflammation, oxidative stress, and fibrosis (Recio et al., 2017; Lopez-Sanz et al., 2018; Opazo-Ríos et al., 2020); 5) the chronic intraocular inflammatory disease (as uveitis) (He et al., 2016; Recio et al., 2017; Ahmed et al., 2018) [very recently, in equine recurrent uveitis (ERU), that is, the only spontaneous model of human recurrent uveitis (Larkin et al., 2020)]; 6) the inflammation in the abdominal aortic aneurysm (AAA), to downstream target genes, and to prevent elastase formation induced by AAA (Bernal et al., 2020); and 7) the severity of skin lesions, autoantibody production, and kidney disease in lupus-associated pathologies (Sharma et al., 2021).

**FIGURE 2:**
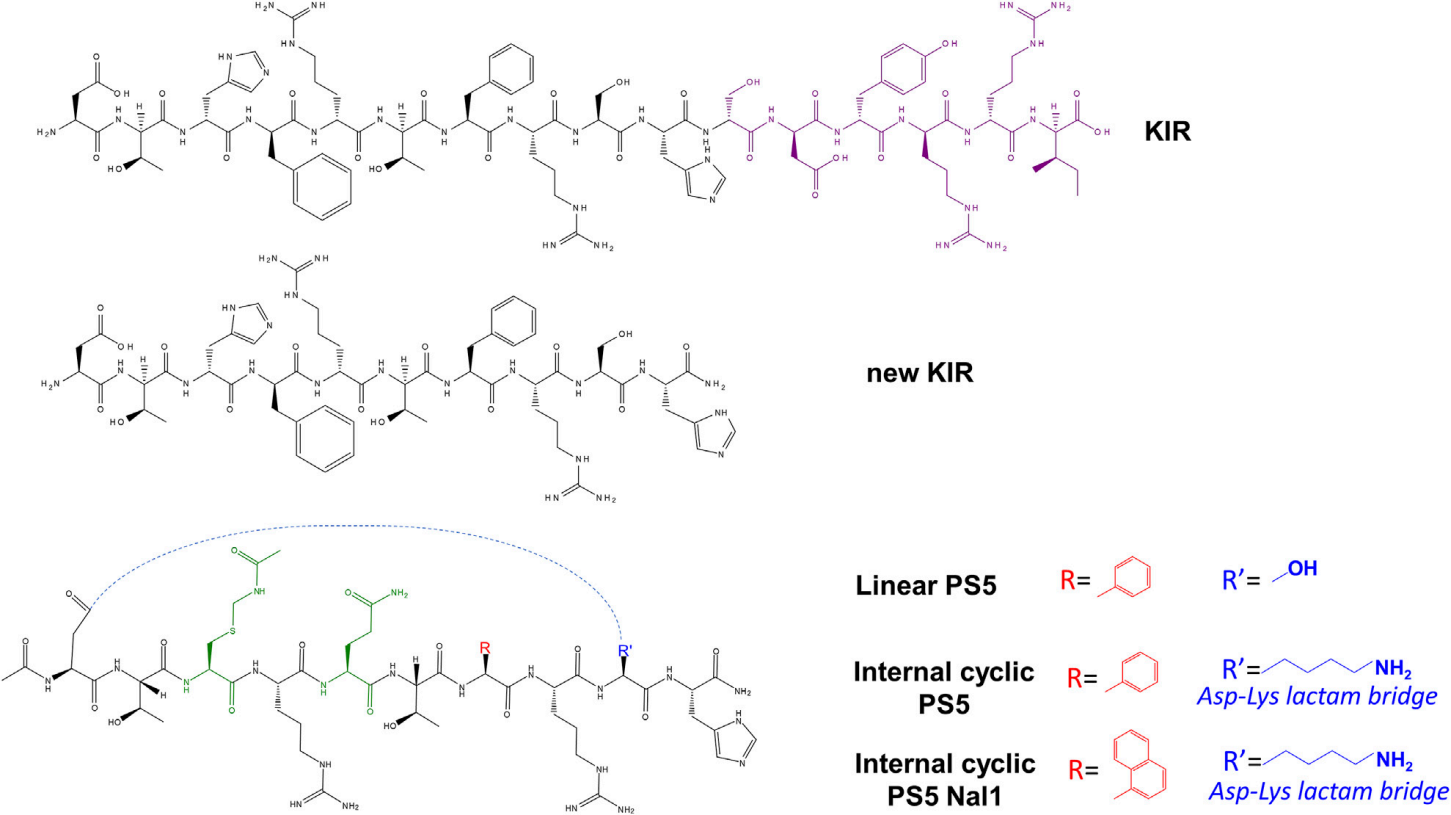
Chemical structures of KIR-based SOCS1 proteomimetics. Green represents non-native residues in PS5 upon combinatorial modifications (Cys(Acm) and Gln), blue represents the residue is alternatively Ser^60^ in PS5 and Lys in internal cycles PS5 for the formation of lactam bridge (with Asp^52^), and red represents the aromatic residue Nal1 or Phe^56^.

In detail, a preclinical study reported the vasculo-protective action of the SOCS1 mimetic KIR peptide in a mouse model of elastase-induced AAA (Bernal et al., 2020). KIR peptide suppressed STAT1,3 activation in aorta, downregulated cytokines and metalloproteinases, altered the expression of cell differentiation markers, and reduced the incidence of AAA in aortic dilation and elastin degradation. Similar effects were encountered in atherosclerotic plaques of diabetic mice where the presence of KIR peptide significantly reduced the size of both early and aged lesions. In addition, the treatment with this SOCS1 mimetic reduced the accumulation of lipids, macrophages, and T lymphocytes and, conversely, determined an increase in the collagen and smooth muscle cell content. The KIR peptide demonstrated its efficacy in an experimental diabetes model with concomitant renal and macrovascular disease (streptozotocin-induced diabetic apolipoprotein E–deficient mouse). Indeed, this compound provided reno- and athero-protection in diabetic mice; in their kidney and aorta, the levels of superoxide anion, DNA oxidation marker, and Nox subunits were significantly lower, while antioxidant enzymes were highly expressed. These results agreed with a concomitant reduction of renal damage (decreased values of albuminuria, creatinine, and tubular injury), atherosclerosis (lesion size), and inflammation (leukocytes and chemokines) (Ortiz-Muñoz et al., 2009). The topical administration of KIR in diabetic retinopathy (DR) reduced glial activation and neural apoptosis induced by diabetes, as well as retinal levels of pro-inflammatory cytokines. These results occurred along with a significant amelioration of electroretinogram parameters, indicating a direct impact on the retina since it also prevented the disruption of the blood–retinal barrier, which was promising for the treatment of early stages of DR (Hernández et al., 2019). One decade ago, through an alanine scan of the KIR sequence of SOCS1, 52–67 fragments, a truncated linear peptide (62–61), named new KIR (Figure 2), demonstrated the ability to bind to JAK2 (Table 2). Subsequently, by carrying out a screening of “combinatorial focused libraries” in positional scanning (PS) format (Marasco et al., 2008; Lonardo et al., 2010; Ponticelli et al., 2008) of new KIR sequence, a new lead compound, named PS5, carrying the mutations His^54^/Cys(Acm), Phe^55^/Arg, and Arg^56^/Gln was investigated (Figure 2). It demonstrated the ability to bind to the JAK2 catalytic site more efficiently than KIR, as evident by the comparison of K_D_ values reported in Table 2, and to drastically reduce STAT1,3 activation induced by IFN-γ and IL-6 and downstream genes (Doti et al., 2012; Madonna et al., 2013; La Manna et al., 2017b). In detail, PS5 effects were analyzed in keratinocytes, and explants of human skin were activated by IFN-γ that have a crucial role in type-1 skin disorders. The inhibition of the phosphorylation of JAK2, IFN-γRα, and STAT1 and the reductions in the gene expression of *IRF-1* transcription factor, *ICAM-1*, *HLA-DR*, *Cxcl10*, and *Ccl2* were observed. Consistently, the adhesiveness and migration of T lymphocytes to autologous keratinocytes were reduced (Madonna et al., 2013). Since both cell migration and proliferation concur to vascular remodeling during the formation of atherosclerotic plaques (Hu et al., 2019), PS5 reduced the migration and proliferation (“wound healing”) of VSMCs. In addition, for both VSMCs and RAW 264.7 cells, PS5 demonstrated antioxidant properties since its presence caused a reduction in intracellular O_2_^•−^ production and the gene expressions of *Nox1* and *Nox4* and simultaneously increased the expression of superoxide dismutase 1 (*Sod1*) and catalase (*Cat*)*.* PS5 also displayed antioxidant and athero-protective properties in a mouse model of atherosclerosis, corroborating the therapeutic perspective for its *in vivo* application. Indeed, PS5 administration to atherosclerotic mice induced a reduction in 1) the size and extension of atheroma plaques, 2) the intraplaque lipid content, and 3) the accumulation of monocytes/macrophages (La Manna et al., 2020b).

**TABLE 2:** SOCS1 proteomimetics: name, sequence, length, and dissociation constants values (µM) toward JAK2 catalytic domain.

Name	Sequence	Length	K_D_ (µM)
KIR	^52^DTHFRTFRSHSDYRRI^67^	16	77^a^
New KIR	^52^DTHFRTFRSH^61^	10	27^a^
Linear PS5	DTC(Acm)RQTFRSH	10	7.0^b^
Internal cyclic PS5	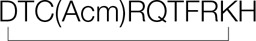	10	44^c^
Internal cyclic PS5 Nal1	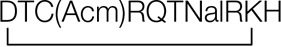	10	35^c^

a ELISA (Doti et al., 2012).

b SPR (La Manna et al., 2017b).

c MST (La Manna et al., 2020b).

In attempts to improve PS5 drug-like features, since no structural details were obtainable for its intrinsic flexibility, cyclic analog compounds were conceived by introducing 1) a disulfide bridge at the extremities (La Manna et al., 2017b), or 2) a lactam bond bridging the side chains of aspartic acid (naturally occurring at position 52) and a lysine residue introduced to substitute Ser^60^, named internal cyclic PS5 (Figure 2). To further enhance the proteolytic stability and the rigidity of new proteomimetics, a non-natural L-1-naphthylalanine in place of Phe^58^ was introduced— internal cyclic PS5 Nal1 compound (Figure 2) (La Manna et al., 2021).

Docking, molecular dynamics (MD), and *in vitro* binding assays suggested that even if van der Waals and electrostatic interactions mainly drive the recognition between JAK2 and internal PS5 cycles, the substitution of Phe/Nal1 allow a greater affinity toward protein target, conferring to aromatic interactions a crucial role for the formation of the complex as also confirmed by structural investigations, circular dichroism (CD), and nuclear magnetic resonance (NMR). In cellular contexts, such as VSMCs and macrophage cell line RAW 264.7, both PS5 cyclic analogs reduce STAT1 cellular migration at a longer extent with respect to Phe-internal cycle PS5, and this could have an impact on ongoing studies of their effects in plaque formation (Charo and Taub, 2011; Demina et al., 2021) as well as for antioxidant properties (La Manna et al., 2020b), confirmed by PCR analysis, which demonstrated a drastic suppression of NADPH oxidase genes and increase in *Sod1* and *Cat*.

Conversely, a small synthetic peptide spanning the phosphorylation activation loop of JAK2, named pJAK2, demonstrated the ability to act as an antagonist of SOCSs. Indeed, in keratinocytes infected with HSV-1, it prevented mice from lethal doses of vaccinia, influenza A, and encephalomyocarditis virus infections (Subramaniam et al., 2004) (Huang et al., 2020). Moreover, during methicillin-resistant *Staphylococcus aureus* (MRSA) skin infection, SOCS1-deficient mice displayed reduced lesion size and bacterial loads and increased abscess thickness when compared to wild-type mice. The treatment of normal mice with pJAK2 increases phagocytosis and bacterial killing, as well as the levels of INFs, restoring skin host defense in hyperglycemic mice (Klopfenstein et al., 2021).

#### SOCS3

SOCS3, by forming a ternary complex, interacts simultaneously with the JAK2 kinase domain and glycoprotein 130 (gp130) phosphotyrosine peptide via two adjacent binding interfaces (Kershaw et al., 2013): on JAK2 Gly^1071^, Gln^1072^, and Met^1073^ (GQM motif) interaction with Leu^22^, Lys^23^, and Thr^24^ of KIR-SOCS3 and Val^34^, Val^35^, and Val^38^ of ESS-SOCS3 that is the most perturbed region upon complex formation (Babon et al., 2006). Additional contacts involve a “hinge” region between ESS and helix A (HA) of SH2, where the stretch 46–52, called CONG, bears three adjacent aromatic residues, FYW. In attempts to obtain SOCS3 proteomimetics, for the first time to the best of our knowledge, we analyzed, in three different studies, the ability of different linear peptides with variable lengths, reported in Table 3, all centered on the KIR domain, to recognize JAK2 and to mimic SOCS3 cellular effects. Initially, we analyzed KIR and ESS regions isolated and in combination: the entire KIRESS region exhibited good affinity for JAK2 and an efficient suppression of both IL-22 signaling in keratinocytes. In detail, the KIRESS region prevents STAT3 and Erk activation induced by IL-22 as well as the expression of STAT3-dependent downstream genes, dampening the proliferative and migratory potential of keratinocytes. In addition, in athymic nude mice bearing squamous cell carcinoma (SCC) xenograft, KIRESS concomitantly reduced the tumor growth and STAT3 levels (Madonna et al., 2017). KIRESS effects were also investigated in IL-6 signaling in mouse xenografts of MDA-MB-231-luci tumors as models of human triple negative breast cancer (TNBC) (La Manna et al., 2018b). The uptake of KIRESS, in 4T1 tumor-bearing BALB/c mice, was more efficient in primary tumor as well as in the bone marrow and spleen compared to non–tumor-bearing mice, allowing an easy access for SOCS3 mimetic to disseminated tumor cells in these organs. Indeed, NOD-SCID animals treated with KIRESS exhibited reduced tumor growth and the complete elimination of pulmonary metastasis as assessed by optical imaging in live mice and *ex vivo* images and histological examination of lungs. The specificity of its MOA was assessed through the evaluation of the SOCS3-pSTAT3-NF-kB pathway by Western blotting experiments. In them, KIRESS significantly reduced the phosphorylation of STAT3 and NF-kB (p65), as well as the expression of inflammatory cytokines including IL-6, leptin, and RANTES in a way similar to the whole SOCS3 protein allowing also its accumulation as consequence of the inhibition of the pSTAT3-NF-kB pathway (La Manna et al., 2018b). More recently, KIRESS peptide demonstrated its efficacy in pathological neovascularization, that is, the major cause of vision loss in the elderly neovascular age-related macular degeneration (nAMD) (Wang et al., 2021). Indeed, the SOCS3 pathway is strictly linked to neovascularization. In a mouse model with a laser-induced choroidal neovascularization (CNV), SOCS3 was significantly induced in myeloid lineage cells; its overexpression inhibited CNV, reduced myeloid lineage–derived macrophage/microglia recruitment onsite, and attenuated pro-inflammatory factor expression; similarly, the presence of KIRESS peptide reduced *in vivo* CNV.

**TABLE 3:** SOCS3 proteomimetics: name, sequence, length, and dissociation constants values (µM) toward JAK2 catalytic domain.

Name	Sequence	Length	K_D_ (µM)
KIR	^22^LKTFSSKSEYQL^33^	12	360^a^
ESS	^34^VVNAVRKLQESG^45^	12	>>1,000^a^
KIRESS	^22^LKTFSSKSEYQLVVNAVRKLQESG^45^	24	6.43^a^
ESSCONG	^34^VVNAVRKLQESGFYWSAVT^52^	19	No binding
restKIRESSCONG	^25^FSSKSEYQLVVNAVRKLQESGFYWSAVT^52^	28	No binding
KIRCONG chim	KβAla^25^FSSKSEYQL^33^βAlaβAla^46^FYWSAVT^52^	20	11.0^b^

a SPR (La Manna et al., 2018b).

b MST (La Manna et al., 2020a).

To explore the contribution derived from other SOCS3 regions, more recently the CONG region, was investigated in a chimeric peptide, named KIRCONG chim, containing non-contiguous fragments: a smaller region of KIR (25–33) and CONG (46–52) connected by β-alanines as spacers (Table 3). This proteomimetic showed a micromolar value of K_D_ and a good α-helical conformation, as indicated by NMR and MD studies. Its anti-inflammatory properties were investigated in VSMC and RAW 264.7 macrophages. The presence of KIRCONG chim suppressed STAT3 phosphorylation and its translocation into the nuclei, as well as the expression of genes as *Cxcl10* and *Ccl5* chemokines and *Nox2*, a superoxide-generating enzyme. The observed anti-inflammatory and antioxidant properties of KIRCONG chim corroborate the potential application of SOCS3 mimetics in inflammatory diseases, including atherosclerosis, even if its low aqueous solubility and high molecular weights hamper their direct use as drugs. Ongoing structure−activity relationship (SAR) investigations through the introduction of ionizable moieties and of conformational constraints confer more suitable drug-like properties (unpublished results).

## Conclusion

The development and clinical and preclinical application of small-molecule JAKis and cytokine-targeted biologics have demonstrated that the inhibition of the JAK-STAT pathway can be an efficacious way to treat inflammatory and autoimmune diseases. The identification of molecular mechanisms by which JAK-STAT signaling is regulated may also reveal opportunities to control signaling in new routes. A recent and interesting application regards the actual pandemic COVID-19 in which the SARS-CoV-2 infection triggers inflammation via the JAK-STAT pathway toward a cytokine storm (Di Natale et al., 2020b). JAKis like ruxolitinib, baricitinib, and tofacitinib were employed to reduce inflammation; several regulatory authorities have supported their use, and numerous clinical trials are in progress to prove their safety and efficacy (Satarker et al., 2021). While the therapeutic efficacy of these inhibitors is unquestionable, an important issue still to be answered is related to their best dosage and safety aspects to meet the requirements of the regulatory authorities. An advancement of JAKis in the drug market is expected on the basis of encouraging preclinical and clinical results. In detail, they are expected to find a role in the emerging therapeutic procedures as high-dose induction, low-dose maintenance, or combination therapies (Satarker et al., 2021). Noticeably, the onset of adverse effects and resistance to some of their treatments under chronic inflammatory conditions outline the need to develop more targeted therapeutics with novel MOAs to overcome these limitations. Indeed, a series of warnings on JAKis are hampered to reach the trial’s primary objectives, in terms of clearances and labeling restrictions (Reddy and Cohen, 2020).

In the last decades, the inhibition of PPIs represents a major challenge in chemical biology, and many drug discovery processes are focused on it. Often peptide-based proteomimetics reproduce the biophysical binding selectivity of large proteins in cellular contexts. Furthermore, there is great interest in developing selective protein kinase inhibitors by targeting different sites with respect to catalytic ones; these involve PPIs that, in general, cannot be addressed with small molecules. The great similarity of ATP-binding sites among kinases partially hampers the identification of selective ATP-competitive inhibitors (Rettenmaier et al., 2014). Conversely, the experimental 3D structure of protein complexes can act as unique templates to design novel and selective modulators of cellular pathways by mimicking protein interfaces. In the case of proteomimetics within the JAK-STAT pathway, they can also provide the required selectivity with respect to the inflammatory mediator that triggers inflammatory response (interleukins, interferons, etc.).

Several investigations of peptides targeting SOCSs or their interactors, conceived as mimetics or antagonists, are crucial starting points for the development of drugs for diseases in which these proteins play a role. Their investigations through biophysical and functional perspectives along with their structural and chemical modifications appear mandatory for the development of novel therapeutic agents.
